# AGREEMENT OF MEDICARE PART D VS. MINIMUM DATA SET REPORTED MENTAL HEALTH DRUGS USE IN NURSING HOMES

**DOI:** 10.1093/geroni/igae098.4285

**Published:** 2024-12-31

**Authors:** Tianwen Huan, Orna Intrator, Adam Simning, Kenneth Boockvar, David C Grabowski, Shubing Cai

**Affiliations:** University of Rochester, Rochester, New York, United States; Icahn School of Medicine at Mount Sinai, New York, New York, United States; University of Rochester, Rochester, New York, United States; University of Alabama, Birmingham, Alabama, United States; Harvard Medical School, Boston, Massachusetts, United States; University of Rochester, Rochester, New York, United States

## Abstract

Researchers have often used the nursing home Minimum Data Set (MDS) data to measure the rate of medication use, but little evidence exists on the accuracy of the MDS-based medication items. We compared quarterly rates of antipsychotic, antidepressant, and hypnotic use between the MDS and Part D Event file (PDE) in 2018 among long-stay nursing home residents enrolled in Medicare Part D who were ≥ 65 years old with psychiatric disorders or dementia identified in the MDS data. We used Cohen kappa to assess agreement in the share of residents using mental health medications during the quarter and used PDE data as a reference to calculate validity parameters. A total of 34.0%/73.4% of study participants were identified using antipsychotic/antidepressant medications in MDS assessments vs 34.3%/71.3% identified in PDE records (kappa value: 0.90/0.83). The sensitivity, specificity, positive predictive value (PPV) and negative predictive value (NPV) of MDS data compared to PDE were 96.7%/84.5%, 92.8% /96.7%, 96.3%/91.1%, and 93.6%/94.0%. Only 3.7% of study participants identified in the MDS data, vs 32.6% PDE-based users (kappa value: 0.1), used hypnotics. By combining anxiety medications with hypnotics, the rates of MDS hypnotic or anxiety users increased to 35.0% (kappa value: 0.74). The sensitivity, specificity, PPV, and NPV were 89.5%, 85.6%, 92.8%, and 79.8%, respectively. Agreement between the MDS and PDE in antipsychotic and antidepressant use was high, suggesting that the MDS is a valid tool to measure antipsychotic and antidepressant use. Additional work is needed to understand the disagreements between MDS and PDE in hypnotic use.

